# Comparing Direct Measurements and Three-Dimensional (3D) Scans for Evaluating Facial Soft Tissue

**DOI:** 10.3390/s23052412

**Published:** 2023-02-22

**Authors:** Boris Gašparović, Luka Morelato, Kristijan Lenac, Goran Mauša, Alexei Zhurov, Višnja Katić

**Affiliations:** 1 Faculty of Engineering, University of Rijeka, Vukovarska 58, 51000 Rijeka, Croatia; 2 Center for Artificial Intelligence and Cybersecurity, University of Rijeka, R. Matejčić 2, 51000 Rijeka, Croatia; 3 Faculty of Dental Medicine, University of Rijeka, Krešimirova 40-42, 51000 Rijeka, Croatia; 4 Clinical Hospital Centre Rijeka, Krešimirova 42, 51000 Rijeka, Croatia; 5 Applied Clinical Research & Public Health, School of Dentistry, Cardiff University, College of Biomedical & Life Sciences Heath Park, Cardiff CF14 4XY, UK

**Keywords:** 3D scanning, 3D surface imaging, dental treatment, functional appliances, oral surgery, orthodontics, soft tissue analysis

## Abstract

The inspection of patients’ soft tissues and the effects of various dental procedures on their facial physiognomy are quite challenging. To minimise discomfort and simplify the process of manual measuring, we performed facial scanning and computer measurement of experimentally determined demarcation lines. Images were acquired using a low-cost 3D scanner. Two consecutive scans were obtained from 39 participants, to test the scanner repeatability. An additional ten persons were scanned before and after forward movement of the mandible (predicted treatment outcome). Sensor technology that combines red, green, and blue (RGB) data with depth information (RGBD) integration was used for merging frames into a 3D object. For proper comparison, the resulting images were registered together, which was performed with ICP (Iterative Closest Point)-based techniques. Measurements on 3D images were performed using the exact distance algorithm. One operator measured the same demarcation lines directly on participants; repeatability was tested (intra-class correlations). The results showed that the 3D face scans were reproducible with high accuracy (mean difference between repeated scans <1%); the actual measurements were repeatable to some extent (excellent only for the tragus-pogonion demarcation line); computational measurements were accurate, repeatable, and comparable to the actual measurements. Three dimensional (3D) facial scans can be used as a faster, more comfortable for patients, and more accurate technique to detect and quantify changes in facial soft tissue resulting from various dental procedures.

## 1. Introduction

Researchers [1,2,3] have been employing various two-dimensional (2D) methods to manage measurements obtained from standard photographs or X-rays in various projections [4,5] or directly from subjects [6,7]. However, the recent development of new acquisition techniques and relevant software has enabled the use of three-dimensional (3D) scans in various areas of dental medicine. These improvements include high-quality motion-fixed image capture to provide better sequential frames with landmark detection. Three-dimensional surface scanning can generate a 3D soft tissue model of the face. The scanning equipment, such as infrared laser digitisers, stereophotogrammetric cameras, or structured-light scanners, is non-invasive [8,9]. At the same time, some researchers employ computed tomography (CT) or cone beam computed tomography (CBCT), which emits radiation [10]. Facial images are usually studied using anatomical landmarks (e.g., see Farkas [11]). Each landmark is defined by three coordinates in the x, y and z spatial dimensions. The set of all landmarks representing a 3D model of the face is known as a landmark configuration or a shape, and such configurations are further analysed using the methods of geometric morphometrics. The development of software to study 3D images tends to create subgroups based on soft tissue shape differences, as opposed to traditional predefined facial characteristics used in 2D studies [12]. The accuracy of the non-invasive facial scanners, usually 0.2 to 1 mm, is satisfactory for clinical purposes [13]. However, there are differences between various scanning techniques and manufacturers [14,15]. Nevertheless, the advancement in scanning technology and computational methods have made non-invasive scanners available at affordable prices, which could facilitate and further promote research and clinical application of 3D models. Additionally, independent initiatives in software development help verify the application of commercially available low-cost equipment. Analysing patients’ soft tissues and the effect of various dental procedures on their facial physiognomy is quite demanding. The operator performing manual physical measurements must possess sufficient knowledge and exhibit considerable caution to the patient. Because there is direct contact between the soft tissue and the instrument, the process can cause discomfort for the patient, especially after certain procedures that may result in swelling and pain. In addition, it is time-consuming for both the patient and the examiner.

To minimise discomfort and simplify the process, we performed facial scanning and computer measurement of experimentally determined demarcation lines. Demarcation lines are virtual boundaries or lines that separate different areas or connect two different anatomical points on the face. Demarcation lines used in this research are chosen based on previous research for the assessment of the post-surgery oedema [4,5,6]. The image acquisition was carried out with a low-cost 3D scanner which requires many consecutive recordings of the object for the best results. RGBD integration was used for merging frames into a 3D object [16]. For correct comparison, the resulting images need to be registered together, which is usually performed with Ransac [17] and ICP [18] based techniques. We used the slower but more precise ICP method [19]. The measurements on 3D images can be performed using exact or approximate distances. With approximate distances, it is assumed that the path between two close points can be approximated by Euclidian distance (neglecting the effect of the curvature). With a more accurate approach, a kind of exact measurement, one subdivides the path into several portions; the algorithm is similar to that of Dijkstra (e.g., see Mitchel and Mount [20]).

This study aimed to evaluate the accuracy and repeatability of facial scans obtained with a low-cost 3D camera and compare the direct measurements from patients’ faces with measurements from 3D facial images for use in dental medicine research.

Hypotheses are:

(1) Three dimensional (3D) facial scans are reproducible with high accuracy;
(2) The actual and computed measurements are consistent and interchangeable.

## 2. Methodology

### 2.1. Data Acquisition and Analysis

Collecting high-quality data is the first step in modelling the face and head. To our knowledge, there are no 3D head scans available in the market with the exact measurement of all facial demarcation lines. We need to measure, capture and create 3D models. To speed up data acquisition and 3D mesh model creation, we used Bellus3D software and Arc scanner (Bellus 3D, version 1.6.2, Bellus3D, Inc., Campbell, CA, USA). The software provides RGB and infrared imaging of extremely detailed facial data. It provides the ability to generate 3D models in the desired ply format. Each generated model has approximately 1,500,000 polygons. To speed up calculations, all models were downsampled to 35,000 polygons using the surface simplification technique [19]. The scanning process involves four different head movements: a left and right rotation to 90 degrees and an up and down rotation to about 45 degrees. Moving the head around its axis is essential for capturing depth information of the face. This is a common procedure known as rgbd integration [16,21]. To prove repeatability, it is necessary to repeat the mapping of the same object more than once. We scanned and overlaid different pairs of head scans to see the differences between the two scans and determine the influence of the scanner. In total, 39 subjects, all attendees of the regional clinical hospital centre, were invited to participate in the study. All participants signed informed consent, and the study was conducted in accordance with the Declaration of Helsinki (1964). Ethical approval was obtained from the local ethical committee (protocol code 003-05/22-1/84). Every subject had two consecutive facial scans taken for further analysis. One operator took physical measurements of reference (demarcation) lines on subjects. These reference lines are usually used in clinical research regarding the influence of oral surgery procedures on facial swelling after surgery [5,22]. The measurements were repeated on 11 subjects two weeks after the first session to test the reliability of the actual measurements by the operator. Another operator independently analysed the 3D facial scans. Another ten subjects (all with full dental class II occlusion) had different set of scans. The first scan was taken in a habitual occlusion (dental class II); the second after the forward movement of the mandible in order to achieve dental class I occlusion. Those scans were compared to detect and visualise the facial change desired during orthodontic treatment.

Differences were processed using IBM SPSS Statistics for Windows, version 24 (IBM Corp., Armonk, NY, USA). Numeric variables, arithmetic means, and standard deviations (SDs) were calculated. Differences between the corresponding linear measurements obtained by the two methods were evaluated with the Bland–Altman test [23].

In addition, the intraclass correlation coefficient (ICC) index was calculated. Values above 0.9 indicate excellent reliability, values between 0.75 and 0.9 indicate good reliability, between 0.5 and 0.75 indicate moderate reliability, and below 0.5 indicate poor reliability [24]. Correlation calculation were performed for the results of the two measurement methods. The t-tests for samples to indicate whether the two samples were comparable in terms of means was conducted, statistical significance was set to *p*<0.05.

### 2.2. Refinement

The first step after data collection and rgbd integration was visualization. High quality visualization is required to determine the differences in the obtained 3D models, but this process requires optimal alignment. The process of optimal alignment of two three-dimensional models in the initial position is called global registration. Global registration is a fundamental problem in shape registration and modeling. Such registration methods do not require information about the initial positions of the observed models. They usually lead to less accurate alignment results and are often used as initialization for local refinement methods. Among local methods, the popular point-to-point Iterative Closest Point (ICP) [25] is mostly used for accurate alignment of models. It attempts to determine the transformation between a point cloud and a reference surface or another point cloud by minimizing the squared differences between the related entities, usually referred to as the reference and target. The reference or source entity (point cloud) is denoted in Equation (1), where *S* represents a set of related points $s_{n}$:

(1) $$S=\left\{s_{1},...,s_{{}_{n}}\right\}.$$

The target point cloud is defined as in Equation (2), where *T* represents a set of associated points $t_{n}$:

(2) $$T=\left\{t_{1},...,t_{{}_{n}}\right\}.$$

Given two points set, the ICP method computes the rigid transformation between them by determining the optimal translation and rotation to minimize the sum of squared errors. It finds the missing pairing between each corresponding point with minimizing distances *E*, see Equation (3) where *R* is the rotation, Nt is the number of target points *T*, $t^{\prime }$ is the translation, $s_{i}$ and $t_{i}$ are the corresponding points from the point clouds *S* and *T*.

(3) $$E\left(R,t^{\prime }\right)=\frac{1}{Nt}\sum_{i=1}^{Nt}{∥s_{i}-Rt_{i}-t^{\prime }∥}^{2}.$$

**Figure 1:**
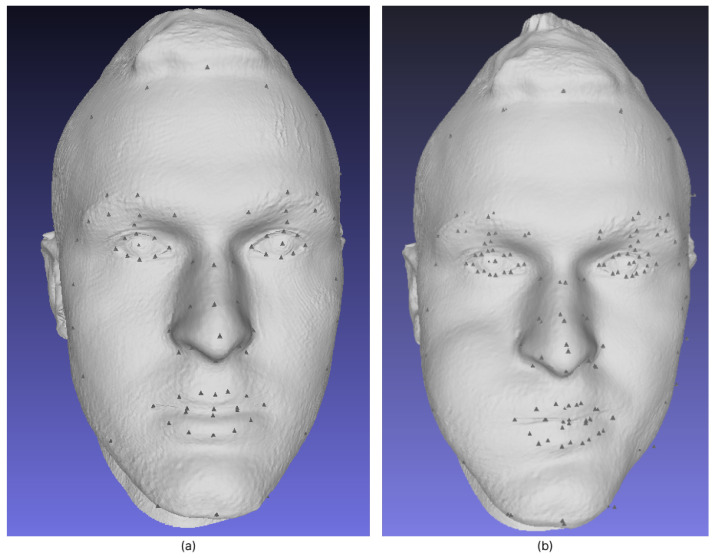
Three dimensional (3D) face mesh with facial landmarks (**a**) Face mesh before operation (**b**) Face mesh after operation.

**Figure A1:**
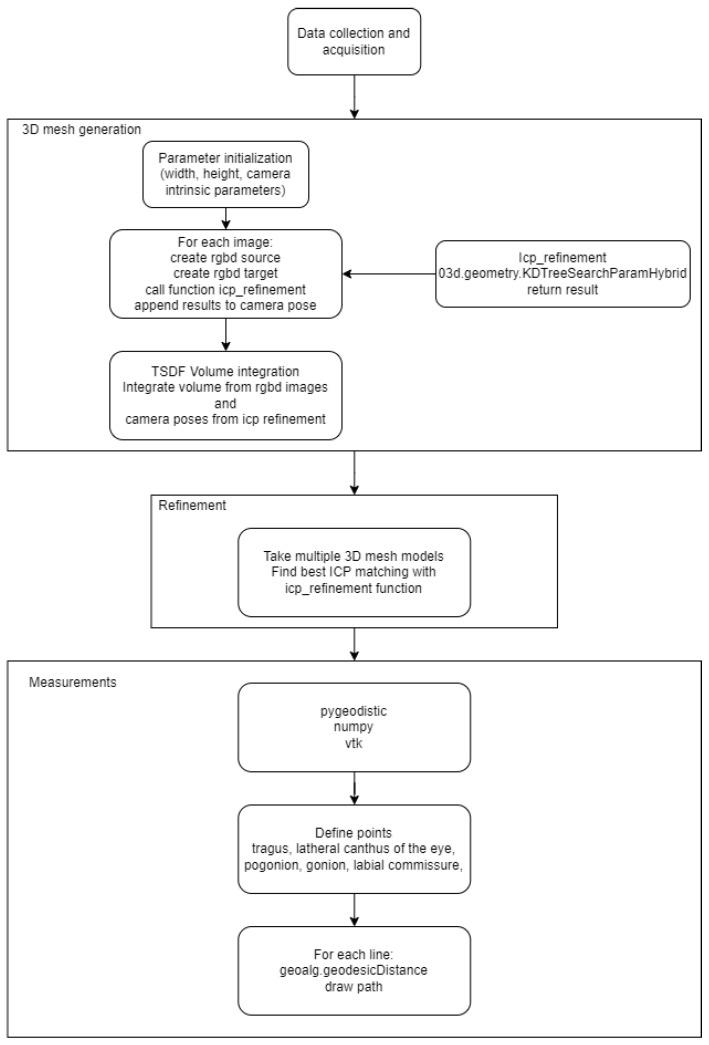
Code flowchart.

The ICP method is commonly used for matching 2D and 3D laser scans, a challenge known as “scan matching”. It has been used in robotics to match scans from 2D laser range scanners [26]. The motion of the robot is proportional to a function that minimizes the difference between two successive snapshots of the environment. Theoretically, it is possible to create a 2D map of the environment by stitching together a series of snapshots. This method can also be used to create 3D maps, but with the disadvantage that errors accumulate between each snapshot. We did not use the ICP algorithm for mapping, but for alignment. We focused on the ICP algorithm to match two repeated head scans of the same object without any changes to it. Although our point clouds consisted of a considerable number of points, it might be useful to use only a few selected points to compute the optimal transformation between two point clouds. It turns out that depending on the data source, some points are more suitable than others because it is easier to find matches for them. For example, if we look at a frontal view of a 3D scanned head model and select known facial landmarks of the face (such as eyes, lips, etc.), it is easy to overlap scanned models. An example of a 3D face mesh with specified landmarks can be found in Figure 1. The left (a) image represents the face before the medical intervention, and (b) image shows an example face after medical intervention (example to study differences, no real interventions were made on this individual). This example has good descriptive features from which it is easy to select individual overlapping points, such as points around the eyes. We chose area and points around the eyes and in the middle of the forehead, because these points remain almost unchanged from childhood to adulthood [11,27]. After selecting important overlapping landmarks, the alignment process becomes simple while reducing the fitting errors for the selected landmarks. Other unselected locations (points) do not contribute to the function that minimizes the difference. For more information on the code workflow, see the Figure A1 in Appendix A.

### 2.3. Mesh Metrics

The faces of all subjects were scanned twice to measure the overlap of the scans. Measurements of the demarcation lines of the face (tragus−lateral canthus of the eye (A); tragus−pogonion (B); gonion−lateral canthus of the eye (C); gonion−labial commissure (D)) were made for each subject (for both right and left side) and compared with the measurements of the same demarcation lines made using the software and automatic positioning of the landmarks. When 3D models are used to study the effects of a particular treatment, the difference can be measured in two ways. First, the difference can be measured on the 3D mesh models, and second, the difference can be measured between 3D scans taken before and after a particular treatment.

#### 2.3.1. Distance on 3D Mesh Model

**Figure 2:**
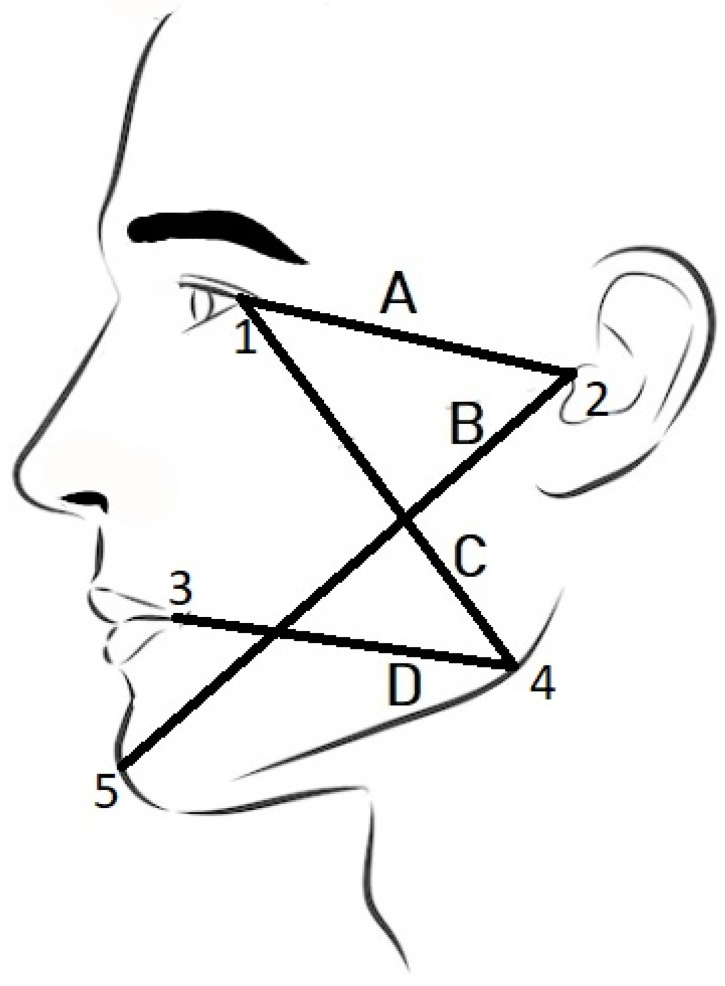
Demarcation lines used for the assessment of the post-surgery oedema; 1—*the lateral cantuhus of the eye*, 2—*tragus*, 3—*labial commissure*, 4—*gonion*, 5—*pogonion*.

The physical measurement of facial changes consists of measuring facial demarcation lines. The demarcation lines are defined by the five critical spots named; latheral cantuhus of the eye, tragus, pogonion, gonion, labial commisure. Figure 2 illustrates the lines formed from all these five points.

Computer measurement of demarcation lines requires the use of 3D images. The three-dimensional image is needed because the measurement on 2D images of defined corners does not provide information about texture and distance from the Z axis distance. Computer measurements are subject to error due to the robustness of 3D depth cameras and mesh modelling algorithms. Despite the initial error, such an approach represents progress in the form of non-contact measurement.

The 3D mesh we created consisted of numerous polygons, or more precisely, triangles. Triangles wer used because they are the simplest two-dimensional objects and GPUs provide very good support for drawing triangles. The distance between two points on a 3D mesh model is called the discrete geodesic problem. It is the shortest path between a source and a destination on an arbitrary polyhedral surface. In this work, we used the implementation of the exact “single source, all destination” algorithm of Mitchel and Mount [20]. The authors used a Dijkstra algorithm [28], a special case called a “continuous” Dijkstra, to find the shortest path to various points over the edges of the surface in the subdivided mesh space. The implementation of such an exact geodesic algorithm for triangular meshes comes from Kirsanov [29]; we used the Cython wrapper for the implemented C++ code.

#### 2.3.2. Distance between 3D Meshes

We chose two methods to determine the differences between two 3D mesh models. The first was the Hausdorff distance, and the second one was the RMSE metric.

Aspert et al. [30] have proposed several metrics for shape distance, of which the Hausdorff distance is the best known. The Hausdorff distance measures the largest distance between two shapes. If we have two shapes (contours) *C* and *D*, we first determine the minimum distance $d_{c}$ between each point *c* on contour *C* and all points *s* on contour *D*, $d_{ps}$ is distance between points, see Equation (4).

(4) $$d_{c}\left(c,D\right)=min\left\{d_{ps}\left(c,s\right),s\subset D\right\}.$$

The second step was to calculate the minimal distance for each boundary point and use the minimal distance with the largest value as the worst-case scenario. See Equation (5), where $d_{c}$ is distance between *c* points and *D* contour points. This metric is not symmetric and $h_{c}\left(C,D\right)\neq h_{c}\left(D,C\right)$, considering that statement. The Hausdorff distance was calculated as in Equation (6), where $H_{C}$ stands for Hausdorff distance, and $h_{C}$ for the worst-case scenario between (*C*, *D*) and (*D*, *C*) distances.

(5) $$h_{c}\left(C,D\right)=max\left\{d_{c}\left(c,D\right),c\epsilon C\right\},$$

(6) $$H_{C}\left(C,D\right)=max\left\{h_{C}\left(C,D\right),h_{C}\left(D,C\right)\right\}.$$

**Figure 3:**
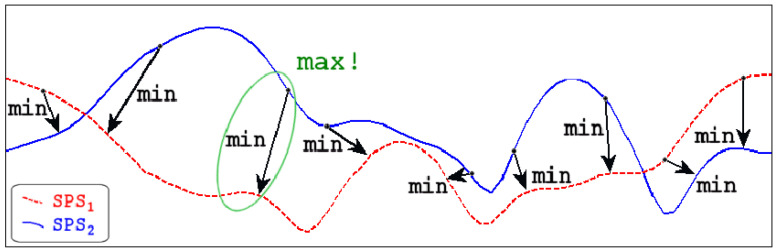
Hausdorff metrics [31].

High-quality meshes typically have numerous vertices and faces, so this calculation is computationally expensive as it is repeated for each link from one point to all the others. A visual representation of the Hausdorff approach is shown in Figure 3:

The root mean square error (*RMSE*) was chosen for comparison of difference results obtained with two measurement methods. See Equation (7), where RMSE stands for Root Mean Square Error Metrics, $ComputerM_{i}$ for the computed measurement, $GroundT_{i}$ for the physical measurement, and *N* for the number of measurements. The relatively low value of the *RMSE* indicates how accurate the model results are.

(7) $$RMSE=\sqrt{\sum_{i=1}^{N}\frac{{\left(ComputerM_{i}-GroundT_{i}\right)}^{2}}{N}},.$$

## 3. Results

### 3.1. Reproducibility and Difference Visualization

Before overlapping and visualizing different head scans, we used an overlap of the repeated same head scan to determine the reproducibility and influence of the scanner. The matching of 39 different pairs of repeated head scans shows results for the Hausdorff distance (min-max) between pairs in the interval [0–46.71] [mm]. The obtained differences show changes in repeated scans in the interval [0.2446, 3.3863][%]. Individuals with longer hair and accentuated hairstyles had the greatest influence on the difference. When we consider the set without such edge samples, we obtain an overlap difference of less than 1% (mean difference of <2 mm), from which we conclude that this is sufficient for further observation of the differences

**Figure 4:**
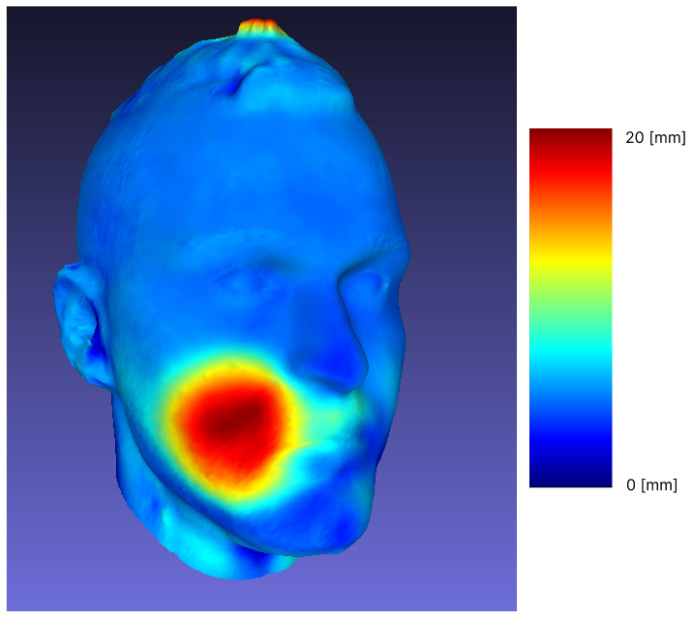
Visualization of differences on 3D meshes.

The highlighting of the differences between two different meshes of the same object consists in the selection of the crucial alignment points and their overlap. The obtained results for the visual identification of object changes are shown in Figure 4 . The differences are shown with the red color channel, and the areas of the red color spectrum were more affected by the outcome of the operations.

### 3.2. Demarcation Lines Measurement

The results for physical (ground truth) and computational measurements (mm) with the corresponding differences (%) between the measurements for the object in Figure 1 are given in Table 1 (example of one sample measurement). The calculation of the difference for each pair was performed as in Equation (8) where $c_{m}$ is the computed measurement and $o_{m}$ is the ground truth (operator measurement).

(8) $$PairDifference=\frac{|c_{m}-o_{m}|}{o_{m}}*100\;\left[\%\right].$$

It should be noted that the physical measurements do not represent the “real” ground truth. Repeated physical measurements showed deviations in the range of 0–10 [mm]. Table 2 shows the ICC indexes for every demarcation line and both right and left sides of the face. Excellent repeatability was achieved for just one demarcation line (line B—both right and left). The other demarcation lines on the right side fall into good (lines C and D) and poor (line A) repeatability. On the left, all three remaining lines (A, C and D) were found to be poorly repeatable. For this reason, the mean values of the repeated physical measurements were used as ground truth values.

The result for the average difference calculated for the whole sample of patients can be found in Table 3. Differences are indicated in percentages.

Although differences of >1% seem a lot, when we express them in millimeters, we obtain deviations in the range of [0.07–15.19] [mm]. We see that the deviation from ground truth for lines A, B and C is <1 cm, which is an acceptable level of measurement accuracy. The deviation in the measurement of the length of line D can be greater than 1 cm, which means a lower accuracy.

**Figure 5:**
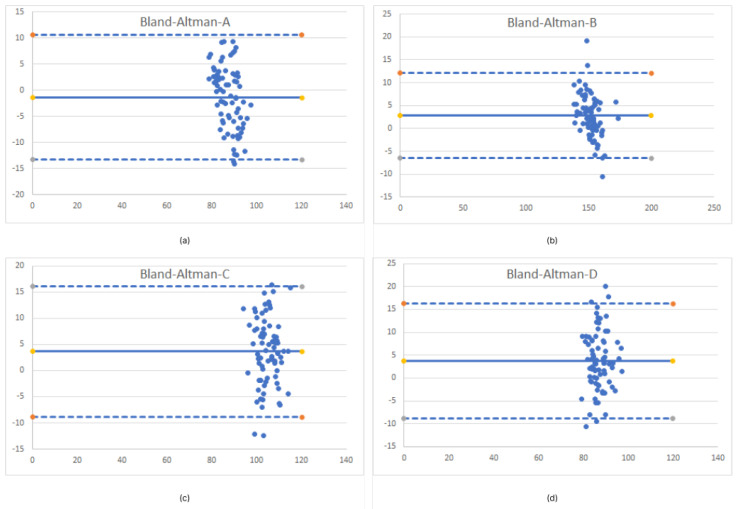
Bland−Altman plots for each demarcation line measurements (**a**)—Limits of agreement for A line (**b**)—Limits of agreement for B line (**c**)—Limits of agreement for C line (**d**)—Limits of agreement for D line.

**Figure A2:**
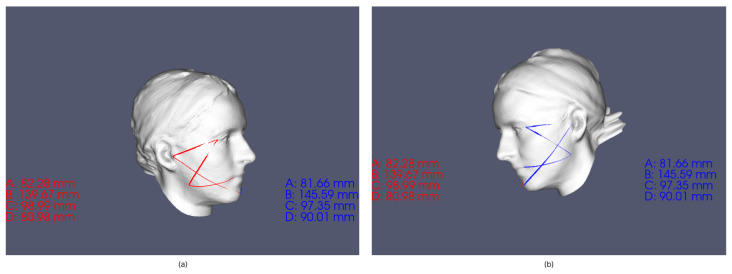
Different camera angles of measurement results (**a**)—Right face view (**b**)—Left face view.

To compare the means of two groups with unknown variances, we also performed a two-tailed t-test (Welch’s t-test). The result for *p* value is 0.31, so we conclude that there is no statistical significance difference between the two groups. Additionally, the correlation between the two measurement groups shows results of 0.973, which shows a strong relationship between the two independent measurement results. To determine the agreement between two measurements, we used the Bland–Altman plot. Ideally, two of our different measurement methods would give the same result, with all differences equal to zero. In a real scenario, there is always some degree of error in any measurement of variables. This approach does not say whether the agreement is sufficient or suitable to use the method. It simply quantifies bias and a range of agreement. The best way to use such a plot would be to define a priori the limits of the maximum acceptable differences, based on biologically and analytically relevant criteria [32]. The limits of agreement are given in the Table 4. Bias is defined as the average value between two measurement methods for each sample. The minimum and maximum limits are a 95% confidence interval for the average difference. Figure 5 represents limits of agreement of two paired measurement methods for every demarcation line. Results from Table 4 and Figure 5 indicate acceptable agreement between the two measurement methods, since the differences are in range [−1.336,3.779] [mm], which is acceptable for the aforementioned clinical research on post-surgery oedema. An example of the results of a computer measurement can be found in the Figure A2 in Appendix A.

### 3.3. Forward Movement of the Mandible

**Figure 6:**
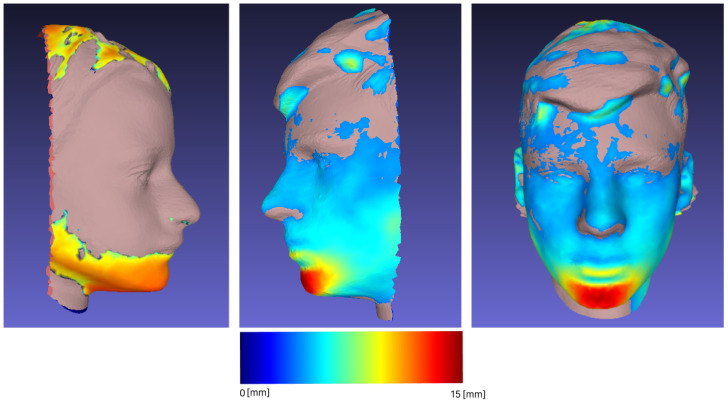
Multiple examples of forward movement.

Additional analyses of the forward movement of the mandible were performed. After overlapping and matching, the Hausdorff distance was calculated and is presented in Table 5. Examples of scan matching (subjects with the most and least detectable changes) are shown in Figure 6. The color scale is composed of RGB channels, where dark red represents a distance of 15 mm and dark blue represents 0 mm. The part without color (shade of brown) is an area viewed at a distance of 0 mm, an aligned domain.

## 4. Discussion

Our research has shown that 3D facial scans from low-cost scanners are reproducible with high accuracy (mean difference between repeated scans < 1%), our initial hypothesis was confirmed. Previous research [13] reported accuracy between 0.2–1 mm for expensive scanners. In contrast, Gibelli et al. [15] found that an inexpensive scanner they tested did not have satisfactory reproducibility (RMS point-to-point distances averaged 0.65 mm), but comparison of the volumes obtained was considered unsatisfactory. In all the aforementioned articles, the scans were manually or semi-manually adjusted before analysis. Usually, the part of the scan that contained the hair was cut off, and landmarks were manually placed on each scan or on each subject before scanning [12,33]. Because these procedures can be time consuming, we attempted to analyse the scans with automated landmark tracking. Automated landmark tracking saves significant time. The tested 3D Bellus software sets a total of over 100 landmarks (ten landmarks for each eye, six landmarks for each eyebrow, fourteen landmarks for the nose, eighteen landmarks for the mouth, ten landmarks for the oblique line of the face, three landmarks for the chin, the trichion hair line, the gonial angle, and the soft tissue throat, and an additional six landmarks for the outer hairline and ten for each ear). As expected, the results became even more accurate when hair was excluded (from initial to after hair removal). Therefore, the use of a hair cover would improve the results without increasing the time needed for the analysis.

The second part of our research deals with the comparison between the computational and the physical measurement of demarcation lines. We chose four lines for measurement, shown in Figure 2, namely *tragus*—*lateral canthus of the eye* (A); *tragus*—*pogonion* (B); *gonion*—*lateral canthus of the eye* (C); *gonion*—*labial commissure* (D). The physical measurements on the subjects showed excellent repeatability only for one demarcation line (tragus-pogonion). It was suggested that this demarcation line could be used in the evaluation of the post-operative swelling of the face, rather than a variety of the demarcation lines [22], because of the long span across the area of the most pronounced swelling. Previous studies have shown that repeatability is better when longer spans are measured [34]. Other demarcation lines showed lower repeatability, which could be due to the effect of facial expressions (slight movements are almost always present, especially in the eye and mouth areas) [35,36]. Additionally, discomfort due to physical contact between the subject’s skin (eye and mouth corners) and the measurement tape must have affected the repeatability; it is also more challenging to place the gonion landmark correctly. Furthermore, measurement of the left side of the face proved even more difficult for the right-handed operator; the poorest repeatability was reported for the demarcation lines of the left side of the face. Any discomfort caused by contact of the tape measure with the sensitive, hypersensitive skin at the corners of the eyes and mouth (resulting in blink reflexes and pinching of the lips) could be avoided by using virtual measurements on the 3D facial scans. However, involuntary blinking and lip curling may still occur during scanning. In these cases, the scanning process should be repeated. To our knowledge, there are no previous reports of measurement error on living subjects who participated in the studies of postoperative swelling after third molar surgery [5,37]. Repeated physical measurement showed deviations in the range of 0–10 [mm]. Although with considerable deviations, we took these measurements as a reference (ground truth). For the exact computational measurement over a triangular mesh, we used the “continuous” Dijsktra method proposed in [20].

Our results for the computational measurement show a difference of <1 cm for demarcation lines A, B, and C. The largest deviation of >1cm from the ground truth measurement is for the D line. The lowest accuracy is limited not only by reflex movement of the lips, but also due to a lower quality selection of the gonion location; rather than a computer measurement error. The gonial angle is a location where the lower mandibular body meets the posterior border of the ramus [38]. Due to the influence of the shape of the face and the amount of fat tissue, it can be challenging to choose an exact gonion position. Considering the experimental results obtained and taking into account the more demanding selection of the gonion location, we can conclude that the computer-based method provides reasonable accuracy. Furthermore, a big part of the measurement error is caused by initial settings. Initial measurement errors are represented by the number of triangles that build a 3D polygonal structure. If the mesh model is created from a smaller number of polygons, the lines that builds the surface area do not describe the real surface curvature. Therefore, it is desirable to have 3D meshes made of a greater number of polygons. Additionally, the precise selection of the source and destination points is also crucial step for computational measurement accuracy. To determine differences between the computational and physical measurements, we performed a two-tailed t-test. The result for the *p* value of 0.31 shows that there is no statistical difference between the two groups and correlation of 0.973 strong association between two measurements. The limits of the agreements are determined for each of the four demarcation lines of the computational measurement. The visualization of such boundaries, here presented with the Bland–Altman diagram (shown in Figure 5), brings us to conclusion that computer-aided measurement provides sufficient accuracy to provide clinically relevant results. The graph shows the less stringent limits for measuring the D line (the aforementioned challenges of actual measurement must be also taken into account, when discussing comparison of the two measurement methods).

In the third part of our study, we examined the procedure of the Frankel manoeuvre (FM). The procedure of the projection of dental class I occlusion in the dental class II patients is called the Fränkel Manoeuvre (FM), and is often used in clinical decision making [39]. If a soft tissue profile becomes more straight (from convex to less convex or straight, but not towards concave), it is advisable to try to gain, as much as possible, forward growth of the mandible with functional orthodontic appliances during the peak pubertal growth [40]. Recording the initial state and comparing it to the projected goal (scanned FM) will enable better evaluation of the end result (after the treatment), also in comparison with the desired outcome (the FM). It is easier to monitor the change with a series of non-invasive 3D facial scans, as opposed to X-ray imaging [4]. The overlap and matching (shown in Figure 6) of different scans of the same individual subjected to FM show a significant change in the forward movement of the mandible, allowing further comparison and tracking of treatment progress, which would not be possible if we were limited to visualization with X-rays. At each follow-up of different dental procedures and for monitoring the changes, the 3D facial scans could be used and measurements could be taken with a minimum of additional time and discomfort for the patients, as the physical contact of the measuring tape with sensitive, prone to reflex contractions and potentially painful parts of the facial soft tissue is reduced.

## 5. Conclusions

The low-cost 3D facial scans are reproducible with high accuracy (mean difference between repeated scans <1%).

The physical measurements are dependent on landmark positioning, sensitivity of the measured area of skin, the measured length and the skill of the operator, but are still considered to be the ground truth; a comparison of computer and physical measurement results shows reasonable accuracy. Facial scans and computed measurements can be used instead of physical measurements.

Further advances for the future use of 3D facial scans are: contactless data acquisition and measuring, better non-invasive visualization in multiple time-points for various dental procedures influencing changes of the facial soft tissues.

## Figures and Tables

**Table 1:** Demarcation lines measurements for Figure 1a.

Line	Physical Distance [mm]	Computational Measurement [mm]	Diff [%]
tragus—lateral canthus of the eye (A)	90.5	88.97	−1.69
tragus—pogonion (B)	163	164.80	+1.10
gonion—lateral canthus of the eye (C)	117	110.46	−5.59
gonion—labial commissure (D)	97	92.85	−4.27

**Table 2:** Intraclass Correlation Coefficient (ICC) for repeated measurements of the demarcation lines (intra-rater reliability).

Line	ICC	
	Left	Right
tragus—lateral canthus of the eye (A)	−0.027	0.435
tragus—pogonion (B)	0.911	0.906
gonion—lateral canthus of the eye (C)	0.423	0.796
gonion—labial commissure (D)	−0.196	0.766

**Table 3:** Difference in distances from ground truth.

Line	Left			Right		
	Diff	Mean [mm]	SD	Diff	Mean [mm]	SD
tragus—lateral canthus of the eye (A)	2.11 [0.79–3.17]	1.60	1.21	1.95 [0.41–3]	1.23	1.29
tragus—pogonion (B)	2.24 [1.29–6.59]	2.694	1.90	1.17 [0.07–2.64]	0.693	1.31
gonion—lateral canthus of the eye (C)	4.44 [0.72–7.52]	3.292	3.69	6.62 [6–10]	7.427	2.08
gonion—labial commissure (D)	10.43 [4.78–15.19]	8.633	5.25	9.60 [4.15–13]	7.940	4.44

**Table 4:** Limits of agreement.

Method Difference	Bias	SD of Bias	Min Limit (95%)	Max Limit (95%)
tr-eye (A)	−1.336	6.077	−13.247	10.575
tr-pog (B)	2.752	4.746	−6.551	12.055
gon—eye (C)	3.637	6.393	−8.893	16.168
gon—comm (D)	3.779	6.391	−8.746	16.305

**Table 5:** Forward movement Hausdorff distance.

Sample	Min [mm]	Max [mm]	Mean [mm]	RMS
1	0	19.59	1.04	1.86
2	0.000259	16.60	2.38	3.24
3	0.000038	10.32	1.39	2.08
4	0.000046	12.98	0.99	1.60
5	0.000015	21.80	1.91	3.26
6	0.000198	20.78	1.85	3.39
7	0	15.55	1.15	2.23
8	0.000031	25.98	2.98	4.77
9	0.000198	11.93	1.26	2.12
10	0.000153	27.86	2.33	4.39

## Data Availability

The data presented in this study are available on request from the corresponding author. The data are not publicly available due to ethical reasons.

## Abbreviations

The following abbreviations are used in this manuscript:

ICP	Iterative Closest Point
RMSE	Root mean square error
ICC	Intraclass correlation coefficient
FM	Fränkel manoeuvre
CT	Computed tomography
CBCT	Cone beam computed tomography

## Appendix A

The Figure A1 provides a comprehensive explanation of the workflow for 3D image registration. This flowchart is divided into three parts. The first part is the 3D mesh generation. We have divided our code workflow into parameter initialization, iteration over images, and the TSDF function. Parameter initialization involves initializing all the important variables, the image width and height, and the camera intrinsic settings (width and height of the camera lens). At each iteration over the dataset, we create the rgbd source and target of the next image and call the icp_function to compute the best parameters for ICP refinement. This function is called *KDTreeSearchParamHybrid* and is provided by the *o3d* library and returns the camera positions. The last part of this phase is the TSDF volume integration. This is a process where different camera poses are connected and a 3D volumetric model is created. This integration is provided by the *o3d* library. The second part of the workflow is defined as refinement. In this step, we use the previous icp_refinement function to find the best ICP results between different 3D mesh models. The last part is the measurement. This script uses the *numpy*, *vtk* and *pygeodistic* libraries. Numpy is used to access the values of 3D meshes loaded with the *vtk* library (function *vtkPLYReader*). The *vtk* library provides a class for 3D visualization with input parameters from the results of the *geodisticDistance* function, which calculates the geodistic distance across the mesh from the source to the target point.

An example of the measurement results on the 3D facial scan is shown in Figure A2. Demarcation lines were measured on both left and right side of the 3D soft tissue facial scans, and compared to the actual measurements of the same demarcation lines on the left and right side of the subject’s face. Each letter marks demarcation lines, as defined in Figure 2.
